# Advances in Multifunctional Materials Obtained at High Temperature and Pressure Conditions

**DOI:** 10.3390/ma19051010

**Published:** 2026-03-06

**Authors:** Lucyna Jaworska

**Affiliations:** Faculty of Metals Engineering and Industrial Computer Science, AGH University of Krakow, Mickiewicza 30 Av., 30-059 Krakow, Poland; ljaw@agh.edu.pl; Tel.: +48-12-617-30-27

The Special Issue “Advances in Multifunctional Materials Obtained at High Temperature and Pressure” comprises a series of articles focused on studying materials obtained from powders. The basic technique for producing materials using powders is sintering. Sintering of ceramic, ceramic–metal, or metallic powders occurs spontaneously at elevated temperatures due to the system’s tendency to lower its surface energy, which decreases when the powder grains coalesce. Materials obtained at high temperatures most often have strong chemical bonds and a high melting point, resulting in a low diffusion coefficient. The 1940s marked the beginning of a scientific approach to sintering. During this era, the first quantitative models were developed, such as the Frenkel neck growth model and the Kingery and Berg shrinkage model, which took into account multiple transport mechanisms [1]. Until the mid-1950s, the most widely used powder sintering method was free sintering (pressureless sintering), which was performed in various atmospheres. Free sintering is still frequently used because it allows the production of large quantities of materials with well-controlled chemical composition in a single process [1]. For superhard powders, static high-temperature sintering (HP-HT) has been used since the mid-1950s [2]. HP-HT sintering can be applied to materials that are stable at high pressure but metastable under atmospheric conditions, such as diamond and cubic boron nitride (cBN). Depending on the method of pressure application, a distinction can be made between uniaxially loaded sintering (Hot Pressing—HP) and hot isostatic pressing (HIP) [3]. Initially, liquids were used as the pressure transfer medium; later, gas-assisted HIP devices were introduced. Currently, powder consolidation is often achieved by using pressure- and/or field-assisted methods (FAST/SPS, HP-HT, related techniques) to control the microstructure and limit grain growth [4,5,6,7,8]. These materials often contain metastable phases (under atmospheric conditions) with unknown properties [9]. Such conditions also reduce the grain size of the resulting materials and reduce their porosity, leading to good mechanical and non-mechanical properties. The most well-known materials obtained using these methods include diamond, cubic boron nitride, carbides, borides, nitrides, some stainless steels, nickel-based alloys, refractory metals (e.g., tungsten, rhenium, osmium, tantalum, molybdenum, niobium, zirconium, and iridium), and their alloys, etc. The development of new sintering techniques and materials, with particular emphasis on improving material properties and reducing environmental impact, is ongoing. Additionally, powder consolidation techniques include the thermal spraying methods. Plasma spraying is a versatile technique that is used to apply coatings and processing powders, with applications ranging from corrosion resistance to thermal protection [10]. The papers that comprise this Special Issue present examples of most powder consolidation methods, which have been appropriately selected for new materials with various applications.

The six included papers address two strong current research axes in materials science:

(a) The design and fabrication of tool materials and coatings for machining and high-load applications;
(b) The optimization of functional (ferroelectric) materials in terms of dielectric properties and losses.

In the background of presented papers, there are two economic and engineering problems:

(a) Supply chain constraints of key raw materials (including W and Co),
(b) Expectations for higher reliability in extreme applications (temperature, friction, and corrosion).

The dominant topics in the presented Special Issue are superhard and hard ceramic tool materials, obtained using various sintering methods.

In tool materials research, presented by Momot et al., there is a continuing industrial need to create difficult-to-machine alloys, especially nickel-based superalloys, with improved performance, reliability, and cost. Existing research emphasizes that the main challenges are thermomechanical loading in the cutting zone, chemical affinity between the tool and workpiece, and rapid tool degradation due to diffusion, oxidation, notch wear, and chipping. This study presents a comparative analysis of cBN-TiN-Ti_3_SiC_2_ obtained using the HP-HT method and the Al_2_O_3_–ZrO_2_–WC system, sintered by the SPS. The materials were validated in a realistic machining plan for the Inconel 718.

Jaworska describes the principles of binder phase selection for high-pressure sintered (HP-HT) polycrystalline diamond (PCD). Most of the binders used (catalytic and non-catalytic metals, carbides, MAX phases, borides/silicides, and “unconventional” binders) are presented. Their work demonstrates why binders facilitate sintering and how they affect diamond degradation at high temperatures.

Hevorkian et al. used a modified SPS technique during the electroconsolidation process. High AC voltage was applied directly to the powder compact, without the use of a pulse generator. Binderless WC was sintered from micro-powders. The mechanical properties of the sintered materials were comparable to those of nano-WC materials.

Konstanty et al. focused on HIP cemented carbides for specific grooving operations, comparing the effect of PVD coatings with different phase compositions, such as AlTiN + AlCrN, TiAlSiN, AlTiN + TiB_2_ and DLC. Cutting tests confirmed that measurable improvements in tool life could be achieved compared to those facilitated by commercial inserts.

Another example of powder consolidation and application, presented by Książek et al., is the production of anti-wear coatings on ductile cast iron. These are Ni-modified Cr_3_C_2_–NiCr coatings created using HVOF 940. This research, which combines microstructure assessment with tribological and electrochemical studies, supports the trend of evaluating coatings not only in terms of their hardness and porosity, but also for their behavior under coupled conditions (friction and corrosive environments).

The regulatory status for lead zirconate and lead titanate (PZT)-based piezoelectric materials classes them as a complex Delegated Directive (EU) 2015/863 (also often referenced as “RoHS III), and they are technically irreplaceable in many advanced applications. Despite these limitations, PZT remains the primary piezoelectric ceramic solution for many actuator and sensor applications, despite the ongoing development of lead-free alternatives, due to its high electromechanical coupling coefficients, high Curie temperature, and parameter stability. Alternative materials often perform poorly in high-power applications. Current research focuses on co-doping strategies (donor/acceptor), phase control and property stability over time (aging, fatigue, and depolarization). A multicomponent PZT system doped with Mn, Sb, Sm, and W, fabricated by conventional powder processing and pressureless sintering, has been investigated by Bochenek et al. The authors investigated the effect of the phase composition of the materials on their microstructural and ferroelectric properties (P–E loops, polarization levels) by changing the relative content of Sm and W.

**Conclusions**

A common theme of these topics is their emphasis on practicality. Most of the papers within this Special Issue described the influence of microstructure on performance tests (e.g., cutting, tribocorrosion) or addressed key issues related to the selection of materials’ chemical composition (e.g., binder phase in PCD and PZT).

With topics ranging from superhard and hard cutting tools (cBN, WC, and polycrystalline diamond) to cermet–ceramic coatings and piezoelectric PZT ceramics, the authors collectively demonstrate that targeted microstructure control remains the most direct tool for improving the actual performance of materials under demanding thermal, mechanical, and chemical conditions.

## Abbreviations

The following abbreviations are used in this manuscript:

HP-HT	High Pressure–High Temperature.
HIP	Hot Isostatic Pressing.
HP	Hot Pressing.
FAST	Field-Assisted Sintering Technology.
SPS	Spark Plasma Sintering.
PZT	Lead zirconate and lead titanate.
DLC	Diamond-like carbon.
PVD	Physical Vapor Deposition.
PCD	Polycrystalline Diamond.
HVOF	High-Velocity Oxygen Fuel (HVOF).

## References

[1] German R.M. (2017). The Emergence of Quantitative Sintering Theory from 1945 to 1955. JOM.

[2] Katzman H., Libby W.F. (1971). Sintered diamond compacts with a cobalt binder. Science.

[3] Hu C., Li F., Qu D., Wang Q., Xie R., Zhang H., Peng S., Bao Y., Zhou Y., Low I.M. (2014). 8—Developments in hot pressing (HP) and hot isostatic pressing (HIP) of ceramic matrix composites. Advances in Ceramic Matrix Composites.

[4] Munir Z.A., Anselmi-Tamburini U., Ohyanagi M. (2006). The effect of electric field and pressure on the synthesis and consolidation of materials: A review of the spark plasma sintering method. J. Mater. Sci..

[5] Biesuz M., Grasso S.V., Sglavo M. (2020). What’s new in ceramics sintering? A short report on the latest trends and future prospects. Curr. Opin. Solid State Mater. Sci..

[6] Li X.Y., Zhang Z.H., Cheng X.W., Huo G.J., Zhang S.Z., Song Q. (2021). The Development and Application of Spark Plasma Sintering Technique in Advanced Metal Structure Materials: A Review. Powder Metall. Met. Ceram..

[7] Jiang R., Torresani E., Cui G., Olevsky E.A. (2021). Proportional Integral Derivative Control in Spark Plasma Sintering Simulations. Materials.

[8] Kruzel R., Dembiczak T., Wachowicz J. (2023). Optimization of Spark Plasma Sintering Technology by Taguchi Method in the Production of a Wide Range of Materials: Review. Materials.

[9] Mazo I., Molina-Aldareguia J.M., Molinari A., Sglavo V.M. (2023). Room temperature stability, structure and mechanical properties of cubic tungsten carbide in flash sintered products. J. Mater. Sci..

[10] Fauchais P. (2004). Under-standing plasma spraying. J. Phys. D Appl. Phys..

